# Safe Subunit Green Vaccines Confer Robust Immunity and Protection against Mucosal Brucella Infection in Mice

**DOI:** 10.3390/vaccines11030546

**Published:** 2023-02-25

**Authors:** Mostafa F. Abushahba, Alexis S. Dadelahi, Emily L. Lemoine, Jerod A. Skyberg, Swati Vyas, Sagar Dhoble, Vinod Ghodake, Vandana B. Patravale, Jeffrey J. Adamovicz

**Affiliations:** 1 Department of Veterinary Pathobiology, University of Missouri, Columbia, MO 65211, USA; 2 Department of Zoonotic Diseases, Faculty of Veterinary Medicine, Assiut University, Assiut 71515, Egypt; 3 Department of Pharmaceutical Sciences and Technology, Institute of Chemical Technology, N.P. Marg, Matunga (E), Mumbai 400019, Maharashtra, India; 4 Laboratory for Infectious Disease Research, University of Missouri, Columbia, MO 65211, USA

**Keywords:** zoonosis, *Brucella*, lipopolysaccharide, saponin

## Abstract

Brucellosis is a zoonotic disease that causes significant negative impacts on the animal industry and affects over half a million people worldwide every year. The limited safety and efficacy of current animal brucellosis vaccines, combined with the lack of a licensed human brucellosis vaccine, have led researchers to search for new vaccine strategies to combat the disease. To this end, the present research aimed to evaluate the safety and efficacy of a green vaccine candidate that combines *Brucella abortus* S19 smooth lipopolysaccharide (sLPS) with Quillaja saponin (QS) or QS-Xyloglucan mix (QS-X) against mucosal brucellosis in BALB/C mice. The results of the study indicate that administering two doses of either sLPS-QS or sLPS-QS-X was safe for the animals, triggered a robust immune response, and enhanced protection following intranasal challenge with S19. Specifically, the vaccine combinations led to the secretion of IgA and IgG1 in the BALF of the immunized mice. We also found a mixed IgG1/IgG2a systemic response indicating evidence of both Th1 and Th2 activation, with a predominance of the IgG1 over the IgG2a. These candidates resulted in significant reductions in the bioburden of lung, liver, and spleen tissue compared to the PBS control group. The sLPS-QS vaccination had conferred the greatest protection, with a 130-fold reduction in *Brucella* burdens in lung and a 55.74-fold reduction in the spleen compared to PBS controls. Vaccination with sLPS-QS-X resulted in the highest reduction in splenic *Brucella* loads, with a 364.6-fold decrease in bacterial titer compared to non-vaccinated animals. The study suggests that the tested vaccine candidates are safe and effective in increasing the animals’ ability to respond to brucellosis via mucosal challenge. It also supports the use of the S19 challenge strain as a safe and cost-effective method for testing *Brucella* vaccine candidates under BSL-2 containment conditions.

## 1. Introduction

Brucellosis, historically known as Malta fever, undulant fever, or Mediterranean fever, is a common bacterial zoonosis caused by different *Brucella* spp. that affects more than half a million people every year worldwide, the majority of which reside in developing countries [1]. 

The disease causes a wide spectrum of manifestations in the affected individuals, ranging from mild undulant fever, night sweats, and fatigue to chronic fatigue, depression, and even affecting bones, joints, and the heart [2]. Of recognized *Brucella* species, *Brucella melitensis* is the most virulent species in humans [3]. Mixed infections of *Brucella melitensis* and *Brucella abortus* have been reported in both small ruminants and humans [4]. Currently, there is no licensed vaccine for human brucellosis [5]. This poses a significant challenge for preventing and controlling the spread of the disease. Additionally, while antibiotic treatment is available, a significant percentage of patients (5–30%) may experience a reactivation of the infection after therapy has been discontinued, which can lead to prolonged illness and additional economic costs [6,7]. 

In animals, brucellosis mainly causes reproductive disorders in affected herds, leading to significant economic losses for the veterinary sector [8,9,10]. The only control strategy for brucellosis in animal herds is test and slaughter, as treatment options for this intracellular pathogen are limited [10]. This highlights the urgent need for effective disease mitigation strategies for both human and animal brucellosis. Licensed RB51, S19, and Rev1 vaccines are useful preventive measures against animal brucellosis [11]. However, they have some drawbacks that may limit their applicability in certain situations, such as limited efficacy, immunological interference for smooth-type vaccines, and residual virulence in animals and humans [11,12,13,14,15]. These limitations strongly underscore the need for new and effective vaccine alternatives that can be used to protect a wider range of animal species. 

It is well-known that mucosal surfaces, such as the gastrointestinal, respiratory, and urogenital tracts, are the first points of contact for many pathogens [16]. *Brucellae* are particularly efficient at using the respiratory mucous membrane as a common portal of entry in both humans and animals [17,18]. Therefore, vaccines that can induce protective immunity at these mucosal surfaces are highly desirable [16]. It has been demonstrated that different types of immune responses to *Brucella* are elicited depending on the route of infection, which supports utilizing vaccine strategies that reflect typical paths to infection [19]. However, most vaccines are administered parenterally, and these vaccines are not as effective at inducing mucosal immune responses [16]. Mucosal vaccines, on the other hand, have the potential to induce both humoral and cell-mediated immune responses at mucosal sites and throughout the body [16,20]. Additionally, the non-invasive, needle-free administration of mucosal vaccines makes them a highly attractive option for vaccination [21]. 

The use of an acellular antigen as a vaccine candidate is one strategy that researchers have been employing to avoid the weaknesses of live vaccines [22,23]. This approach involves using only a specific part of the pathogen, rather than the whole organism, to stimulate an immune response. This can reduce the risk of side effects and improve the safety of the vaccine, yet they are less immunogenic [22]. 

The present study evaluated the safety and efficacy of a green vaccine made of two plant-derived components, saponin from Quillaja Bark and xyloglucan from tamarind seed, in tandem with *Brucella abortus* S19 smooth lipopolysaccharide (sLPS) against mucosal brucellosis in BALB/C mice. The *Brucella* smooth LPS is several hundred times less toxic and non-pyrogenic compared to other lipopolysaccharides [24], making it a potential alternative vaccine. Its utility in protecting against brucellosis has been demonstrated [24,25,26,27]. Xyloglucan is a natural plant polysaccharide and non-toxic food additive with mucoadhesive and immunopotentiation properties [28,29,30]. Saponins are a class of natural compounds with potent immune-modulating properties, making them a promising candidate as adjuvants in vaccine development [31,32]. Saponins can influence the immune response towards a Th2-type response or elicit mixed Th1/Th2 responses depending on their chemical structure [31] and can improve antigen presentation to T cells and increase phagocytosis and T cell memory response [33,34]. 

Previous studies have shown promising results for the use of mucosal vaccines against brucellosis in mouse models [21,35,36,37,38], and the present study adds to this body of literature by demonstrating that a specific subunit vaccine combination confers enhanced immunity and protection against mucosal *Brucella* infection in mice. These findings suggest that mucosal vaccines may be an effective strategy to enhance resistance to brucellosis infection and may prove to be important for disease eradication programs and preparedness against potential future biological attacks involving aerosolized *Brucellae*.

## 2. Materials and Methods

### 2.1. Chemicals, Reagents, and Antibodies

Phenol solution (90%), Quillaja Saponin (Catalog# S2149), silver nitrate, sodium carbonate, sodium bicarbonate, sodium acetate, Tween 20, DMSO, fetal bovine serum, and casein were obtained from Sigma Aldrich. Tamarind seed polymer (Xyloglucan, Glyloid 2A) was purchased from Sumitomo Pharma, Japan. Laemmli Sample Buffer (2×) and Precision Plus Protein Dual Color Standard were purchased from Bio-Rad. Trichloroacetic acid, 99%, Bovine Serum Albumin Standard Pre-Diluted Set, Coomassie R-250, and phosphate-buffered saline were purchased from ThermoFisher. TMB ELISA Substrate and TMB stop (Abcam, Cambridge, UK), cross-adsorbed goat anti-mouse IgA, IgG1, and IgG2a, HRP-conjugated (Invitrogen, Waltham, MA, USA), ExpressPlus PAGE Gel, 4–20% (Genscript, Piscataway, NJ, USA), Thiazolyl blue tetrazolium bromide (MTT, Trenton, NJ, USA), 98% (Acros Organics, Geel, Belgium) and Brucella Selective Supplement (Oxoid) were used in this study.

### 2.2. Smooth LPS Extraction

sLPS of *B. abortus* S19 was extracted using the warm phenol-water method [39]. Briefly, bacterial cultures of S19 were grown in brain heart infusion broth for 48 h at 37 °C with shaking, centrifuged at 10,000× *g* for 15 min at 4 °C. Bacterial pellets were suspended in a hot distilled water prewarmed to 66 °C, followed by the addition of an equal volume of phenol solution (90% *w*/*v*) and stirring at 66 °C for 20 min. After immediate cooling of the mixture on ice, it was then centrifuged at 4 °C (13,000× *g* for 15 min) and filtered via Whatman paper #3. The filtered-bottom phenol layer containing the smooth LPS was then transferred to a new centrifuge tube. To precipitate the sLPS, 3 volumes of cold methanol containing 1% methanol saturated with sodium acetate were mixed with the solution and maintained at 4 °C for 1.5 h. Sterile distilled water was then used to produce the LPS samples in a solution, followed by adding trichloroacetic acid to remove the protein contaminants and centrifugation at 10,000× *g* for 15 min. The supernatant was then dialyzed against sterile water for 48 h with changing the solution 4 times. The purified sLPS was lyophilized and kept at −20 °C until used.

### 2.3. sLPS Characterization and Analysis

The extracted sLPS was separated on SDS-PAGE and visualized after staining with silver as described [40] with few modifications. Briefly, 20 µL LPS (15 or 20 µg) in PBS was mixed 1:1 with 2× Laemmli Sample Buffer, boiled for 5 min, and loaded into 4–20% ExpressPlus PAGE Gel. Electrophoresis was performed in a Bio-Rad Mini-PROTEAN Tetra system with 1× Tris-MOPS-SDS buffer, at 12 mA in stacking gel and 25 mA in resolving gel. Run was stopped when marker (Precision Plus Protein Dual Color Standard, 10–250 Kda) reached the bottom of the gel. After electrophoresis, the LPS in the gel was oxidized using periodic acid (0.7%) in 40% ethanol–5% acetic acid solution for 20 min at room temperature before being washed 3 times with distilled water. The gel was then stained for 10 min with a solution produced from ammonium hydroxide, sodium hydroxide, water, and silver nitrate. After washing, the color was developed by reduction in water containing citric acid and formaldehyde. Finally, the color reaction was stopped by 10% acetic acid for 1 min and the gel was photographed.

Coomassie blue staining was further used to ensure absence of protein contaminants in the purified sLPS. To this end, five replicates of the characterized *B. abortus* S19 sLPS samples (50 µg each) were run in 4–20% ExpressPlus PAGE Gel at 140 V. Varying concentrations (10, 5, 2.5, and 1.25 µg) of bovine serum albumin and a Precision Plus Protein Dual Color Standard were used as a positive control and a marker, respectively. PBS was served as a negative control. After 45 min electrophoresis, the gel was stained following the manufacturer’s instructions in a staining buffer consisting of 0.1% Coomassie R-250 in 40% ethanol–10% acetic acid solution, and heated in a microwave for 45 sec followed by gentle shaking on an orbital shaker for 15 min. The gel was then destained in 10% ethanol–7.5% acetic acid solution and heated again for 45 s before shaking on an orbital shaker until the desired background was achieved.

### 2.4. Cytotoxicity Assay

To assess the cytotoxicity of various components included in the proposed vaccine candidate, an MTT assay was followed [41] with modification. Briefly, saponin, starting at 120 µg/mL and xyloglucan starting at 800 µg/mL with subsequent two-fold serial dilutions were added to 80% confluent Vero 81 cells in 96-well tissue culture plates. Following dilution, sLPS was added at a fixed concentration of 50 µg/mL to all wells representing the “sLPS-QS or sLPS-QS-X”. PBS was used as a negative control. Cells were incubated with the compounds for 24 h in CO_2_ incubator at 37 °C, then the microtiter plate was washed off and MTT was added at a final concentration of 0.5 mg/mL and kept in the CO_2_ incubator for another four hours before dissolving the formazin with DMSO and measuring the optical density at 595 nm using SpectraMax Plus reader.

### 2.5. Hemolysis Assay

QS has been shown to cause a dose-dependent hemolysis of human red blood cells (RBCs) starting from 25 μg/mL [42] and Wistar rats RBCs at lower doses [30]. Therefore, in this study, we assessed its in vitro hemolytic activity against mice erythrocytes to determine a safe dose for use in our vaccine formulations. A published protocol was followed [43] with few modifications. Briefly, 2 mL of blood from two adult CO_2_-euthanized mice was collected via cardiac puncture into a 15 mL centrifuge tube containing 80 µL, 6.32 USP units per mL, of BD sodium heparin and mixed gently with 5 mL PBS. The sample was then centrifuged at 200× *g* for 10 min using a Sorvall ST 16 Centrifuge (Thermo Scientific) adjusted to 4 °C. The pellet was carefully resuspended in 2 mL of PBS and washed three times in 2 mL PBS each. A 4% solution of the pellet in PBS was prepared, of which 75 μL was gently mixed in 1.5 mL tubes in triplicate with equal volumes of saponin samples pre-adjusted to a concentration range of 120 μg/mL to 15 μg/mL in PBS. The samples were incubated at 37 °C for 30 min and then pelleted using Eppendorf 5417C Digital Centrifuge set at 80× *g* for 10 min. The supernatant (125 μL) was then carefully collected from each tube and transferred to a flat-bottomed 96-well plate. The content of free hemoglobin in the supernatant was measured at 450 nm using SpectraMax Plus reader. PBS and 1% Triton-X-100 were used as controls. The degree of hemolysis induced by each concentration of saponin was calculated relative to the controls, PBS (0% hemolysis) and Triton-X-100 (100% hemolysis).

### 2.6. Vaccine Formulation

The three vaccine components, sLPS, xyloglucan, and saponin, were prepared with final concentrations of 8 mg/mL, 10.66 mg/mL, and 1.6 mg/mL, respectively, using a sterile PBS solution (sPBS) prewarmed to 75 °C. The sLPS suspension was vortexed and kept in a heat block at 75 °C for 5–7 min, while the xyloglucan suspension was thoroughly vortexed and kept at the same temperature for 12 min with vortexing every 2 min to ensure complete dissolving. Safety assessment studies were conducted on the vaccine components as described earlier, and a final non-toxic dosage used to vaccinate mice was 50 µg of sLPS, 15 µg of saponin, and 100 µg of xyloglucan per animal. To achieve these concentrations, saponin was mixed with xyloglucan in a ratio of 1:1 to obtain final saponin and xyloglucan concentrations of 0.8 mg/mL and 5.33 mg/mL, respectively. A volume of 18.75 µL of this mixture was then mixed with 6.25 µL of sPBS for each animal in the QS-X group or with 6.25 µL of sLPS (50 µg) for each animal in the sLPS-QS-X group. For the sLPS-QS group, saponin was mixed with sPBS (1:1) to obtain a final concentration of 0.8 mg/mL, and a volume of 18.75 µL of the saponin–sPBS was mixed with 6.25 µL of sLPS per animal. The vaccine was formulated as needed per each respective group with an extra 10% volume. A sterile PBS (sPBS) group served as a negative control. All vaccine formulations were prepared just before the immunization under complete aseptic conditions and were kept at 4 °C for a short period before use.

### 2.7. Mice Immunization and Challenge

**Figure 1:**
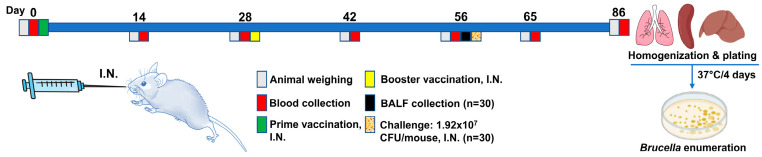
Chart showing the experimental details used in this study. Sixty BALB/C mice were vaccinated at 0 and 28 days. Blood collection was performed using the Lancet method at all the indicated time points except at necropsy day, where cardiac puncture was used. On day 56, BALF samples were collected from 30 mice and the rest of the animals were challenged intranasally using *B. abortus* S19 at 1.92 × 10^7^ CFU/mouse before termination of the study on day 86. Different organs were sampled, homogenized, ten-fold serially diluted, and plated on Brucella selective agar. CFUs were enumerated at day 4 postincubation at 37 °C in a CO_2_ incubator. This chart was created by powerpoint using images and cartoons obtained from clipart-library.com and biorender.com.

In this study, female BALB/C mice, 6–7 weeks old, were purchased from Jackson laboratories and acclimatized to the housing conditions for 1 week before being divided into 5 groups of 12 mice each. The sample size was determined using the Resource Equation (E = Total number of animals−Total number of groups) [44] that indicated an N of 6 animals per group was sufficient to ensure power. For immunization, a dose of 25 μL of vaccine formulation or sPBS was administered dropwise into the nostrils of isoflurane-anesthetized mice. The immunization regimen consisted of two doses given at day 0 and day 28. Four weeks postvaccination, anesthetized mice were intranasally challenged with a specific dose of 1.92 × 10^7^ CFU *Brucella abortus* S19 per animal in 25 µL of sterile PBS (Figure 1). This dose was determined through a ten-fold serial dilution and plating on Brucella selective agar. The challenge dose was used based on a recent study that designated *Brucella abortus* S19 as a challenge strain for a mouse model of brucellosis [45]. All studies were conducted in accordance with the University of Missouri Animal Care and Use Committee guidelines.

### 2.8. Blood and Bronchoalveolar Lavage Sampling

In this study, the Lancet method [46] was used to collect 50–100 µL peripheral blood samples via the submandibular vein at various time points (D1, D14, D28, D42, D56, and D65). Additionally, terminal blood samples were collected from CO_2_-euthanized mice via cardiac puncture on day 86. The collected blood samples were centrifuged at 1500× *g* for 15 min to obtain serum samples, which were then stored at −80 °C. Moreover, bronchoalveolar lavage fluid (BALF) samples were obtained by lavaging the airways of thirty CO_2_-euthanized mice (6 animals per each group) with 1 mL sterile PBS each using 20-gauge regular bevel needle (BD Precision) at day 56 of the study. The collected samples were immediately stored at −80 °C until analyzed by ELISA.

### 2.9. Serology

An endpoint ELISA was used to monitor the antibody titer throughout the study. The assay was primarily optimized using control sera from BALB/C mice challenged intraperitoneally with 1 × 10^6^ CFUs of *Brucella abortus* S19. In the present study, serum samples were tested for the presence of anti-LPS antibodies by ELISA using Clear Flat-Bottom Immuno Nonsterile 96-Well Plates (ThermoFisher Scientific, Rochester, NY, USA). Plates were coated with S19 sLPS at 2.22 µg/mL and 0.3 µg/mL for the IgG2a and IgG1 assays, respectively, maintained overnight at 4 ⁰C, then washed four times with 0.01% Tween in PBS (PBST) and blocked with blocking buffer containing PBST and 3% casein for 15 min at room temperature. Plates were then incubated with serial dilutions of serum (starting dilution was optimized for each time point) from individual animals diluted in blocking buffer and allowed to incubate at 37 °C for 2 h. Plates were washed with PBST and allowed to incubate with goat anti-mouse IgG1 or IgG2a diluted in blocking buffer at 1:1000 or 1:3000, respectively, and allowed to incubate at room temperature in the dark for 1 h. Plates were then washed four times with PBST before the addition of 100 µL of TMB substrate to each well and incubation in the dark at room temperature for 15 min. Finally, 100 µL of TMB stop solution was added to each well before plates were read at 450 nm. A similar protocol was used to determine the level of the IgG1 and IgG2a in the BAL samples; however, BAL samples were used at 1:2 initial dilutions with subsequent two-fold serial dilutions. To determine the IgA levels in BALF samples, 1 µg/mL sLPS was used to coat the ELISA plate and the cross-adsorbed goat anti-mouse IgA HPR conjugate was used at a final dilution of 1:2000. Each sample was run in duplicate and the cut-off was calculated as mean +3 standard deviation of negative controls [47].

### 2.10. Bacterial Enumeration

The spleens, lungs, and part of the liver were sampled from CO_2_-euthanized mice on day 86 of the study, one-month postchallenge, under aseptic conditions. As reported [48], organs were transferred to 2 mL tubes containing 900 mL sPBS and two metal beads before being mechanically homogenized, ten-fold serially diluted, and plated in triplicates onto Brucella selective agar. The number of colonies formed were counted on the 4th day after incubation at 37 °C in a CO_2_ incubator.

### 2.11. Statistical Analysis

The data obtained in this study were analyzed using One-way ANOVA in the GraphPad Prism software 9 (GraphPad Software, Inc., La Jolla, CA, USA). To determine the statistical significance of the differences found between the groups, Fisher’s Least Significant Difference (LSD) test was used as a post hoc test [49]. A *p*-value of less than 0.05 was considered statistically significant.

## 3. Results

### 3.1. Characteristics and Purity of the Extracted LPS

**Figure 2:**
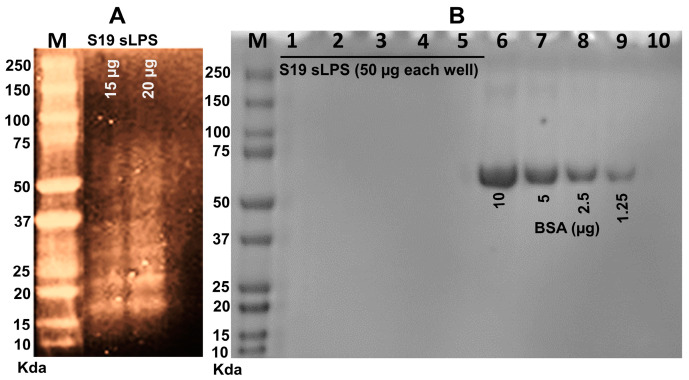
Electrophoretic profile of the purified *B. abortus* S19 sLPS. (**A**) Silver-stained SDS-PAGE image showing the S19 extracted sLPS, 15 and 20 µg, in a smeary appearance. (**B**) Coomassie-stained SDS-PAGE showing absence of protein contaminants in 50 µg LPS samples (Lane 1–5), detectable BSA bands as a positive control (Lane 6–9), and PBS as a negative control (Lane 10). A 10–250 Kda Precision Plus Protein Dual Color Standard (M) was used in (**A**,**B**).

In this study, we extracted *Brucella abortus* S19 sLPS using the warm phenol-water method [39], a popular technique for extracting LPS from various pathogens. We then visualized the purified *Brucella abortus* S19 sLPS by silver and Coomassie staining after separation on SDS-PAGE. As revealed in Figure 2A, the purified sLPS had a characteristic smeary appearance with different band zones of varying molecular weights as expected for the S19 smooth LPS [25]. In agreement with previous research [50], Coomassie blue staining confirmed the purity of the warm phenol-water purified sLPS as no protein bands were observed in five replicates of the purified sLPS (50 µg each), while BSA bands were visible at all tested concentrations, 1.25–10 µg (Figure 2B). This indicates that the extraction was successful and the resulting sLPS was pure and suitable for further experimentation.

### 3.2. In Vitro and In Vivo Assessment Studies Ensured Safety of the Vaccine Components

**Figure 3:**
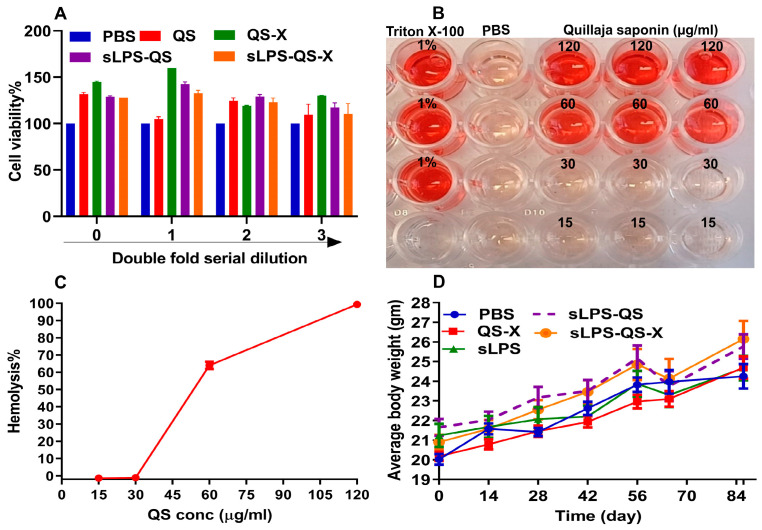
Safety profile of the vaccine components. (**A**) MTT assay showing the impact of different components on the *Vero* cells 81 viability; 0 represents the highest concentrations (120 µg/mL for QS and 800 µg/mL of xyloglucan) that were two-fold serially diluted through 1 to 3. After dilution, a fixed dose of sLPS (50 µg LPS) was added in the indicated groups. (**B**) Hemolytic effect of different concentrations of Quillaja saponin on mouse RBCs in triplicate; 1% Triton X-100 and PBS were used as positive and negative controls, respectively. (**C**) The optical densities of the free hemoglobin shown in (**B**) were determined and hemolysis percentage of different saponin concentrations was calculated relative to the controls. Data represented as average percentage ± SE (A & C). (**D**) Average body weights of different animals throughout the 86-day study period with no significant changes in body weights. Data represented as average body weight ± SE of sixty mice (D0–D56) and as average body weight ± SE of thirty mice (D65 and D86).

The safety of the components included in the current vaccine was ensured by performing an MTT assay of different vaccine components in *Vero* cells and by evaluating the hemolytic properties of Quillaja saponin on mouse RBCs. This cell line has been utilized previously in assessment of the toxicity properties of different saponins [41]. The data from our study indicate that none of the components affected cell viability, even at higher concentrations of 120 µg/mL and 800 µg/mL of saponin and xyloglucan, respectively, when applied individually or in combinations against *Vero* cells, as shown in Figure 3A, indicating a safe profile for the suggested vaccine. Additionally, our in vitro assessment of saponin on mouse RBCs showed that while 120 and 60 µg/mL concentrations induced 99.34 ± 1.15% and 63.97 ± 2.11% hemolysis, respectively, zero hemolysis was detected at 30 and 15 µg/mL (Figure 3B,C).

To further confirm that our vaccine components are safe in vivo, we conducted a small pilot study using two BALB/C mice. In this pilot study, one animal was given 15 µg QS and the other one was given a QS-X mix that included 15 µg QS plus 100 µg xyloglucan. The doses were administered intranasally in 25 µL sterile PBS. The two animals tolarated the QS and the QS-X mix as measured by clinical observation and the absence of weight loss. The two animals were observed for at least two months after the administration with no evidence of toxicity (data not shown). Based on the in vitro and in vivo assessment studies, a dose of 15 μg of QS was considered safe for in vivo experimentations in BALB/C mice. The vaccine efficacy study provided further evidence of the safety of the tested vaccine formulations as all animals survived until the scheduled termination days (day 56 and day 86) with no signs of toxicity and no significant changes in body weight (Figure 3D). 

### 3.3. Qs and Qs-X Modulate the Kinetics of Humoral Immune Responses to Brucella sLPS

**Figure 4:**
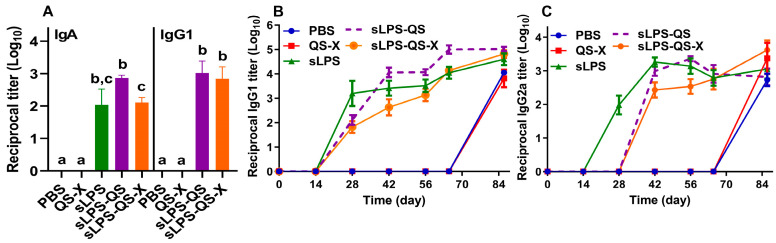
Endpoint titers (Log_10_) of local and systemic antibody responses in different groups: (**A**) Average (Log_10_) IgA and IgG1 anti-LPS titers in mice bronchoalveolar lavage. (**B**) Average Log_10_ of reciprocal titers of IgG1 anti-LPS titer in peripheral mouse serum. (**C**) Average Log_10_ of reciprocal titers of IgG2a anti-LPS titer in peripheral mouse serum. Dates indicate times of sample collection postvaccination. Day 0 and 28 (dates of vaccination), day 56 (day of the challenge), and day 86 (study termination). Data represented as average Log_10_ ± SE. A significant increase in the serum IgG1 and IgG2a levels for all tested groups compared to the PBS or the QS-X was found. Data were analyzed by ANOVA and Least Significant Difference (LSD) was used to determine differences between the groups. Different letters indicate that values are significantly different. A *p*-value of less than 0.05 was considered statistically significant.

To explore whether intranasal administration of our vaccine formulations could induce mucosal immune responses, we assessed the levels of IgA, IgG1, and IgG2a production in the bronchoalveolar lavage fluid of 50% of animals (*n* = 30) at day 28 post boosting. The results showed significant IgA production in all groups where sLPS was used, but not in PBS or QS-X (Figure 4A). The data suggest a possible role of the QS and QS-X in directing the local respiratory immune response against the sLPS as IgG1 was only detected in groups where either QS or QS-X was incorporated with sLPS (Figure 4A). However, no IgG2a was detected in any of the groups.

In this study, we also assessed the kinetics of the systemic humoral response by measuring antibody levels in the sera of the study groups at various intervals before and after being challenged. The results in Figure 4B showed that all treatment groups exposed to sLPS produced specific IgG1 antibodies around day 28, corroborating previous studies which have indicated that sLPS is sufficient to induce *Brucella*-specific antibody responses. The study also found that one treatment group (sLPS-QS) had an enhanced IgG1 antibody response starting 14 days after receiving a second dose of the vaccine. This increase was sustained throughout the rest of the study and was pronounced at day 65 with an 8.98-fold increase relative to sLPS-only vaccination. This suggests that the presence of QS in the vaccine may have played a role in increasing the production of the anti-*Brucella* sLPS IgG1 antibodies and potentially enhancing the overall immune response relative to sLPS vaccination alone. As shown in Figure 4C, all three LPS-vaccinated groups also demonstrated an IgG2a response, though with altered kinetics for groups having the QS or QS-X. No anti-LPS response was detected in either control group (QS or QS-X) at any time point before the challenge or at day 9 postchallenge, but both groups made IgG1 and IgG2a 30 days postchallenge, indicating that the intranasal challenge was successful. Overall, the results suggest that the addition of QS and QS-X to sLPS-based vaccines may play a role in modifying the kinetics of the immune response against the sLPS.

### 3.4. Qs and Qs-X in Tandem with sLPS Enhance Protection against Mucosal Brucella Infection in Mice

**Figure 5:**
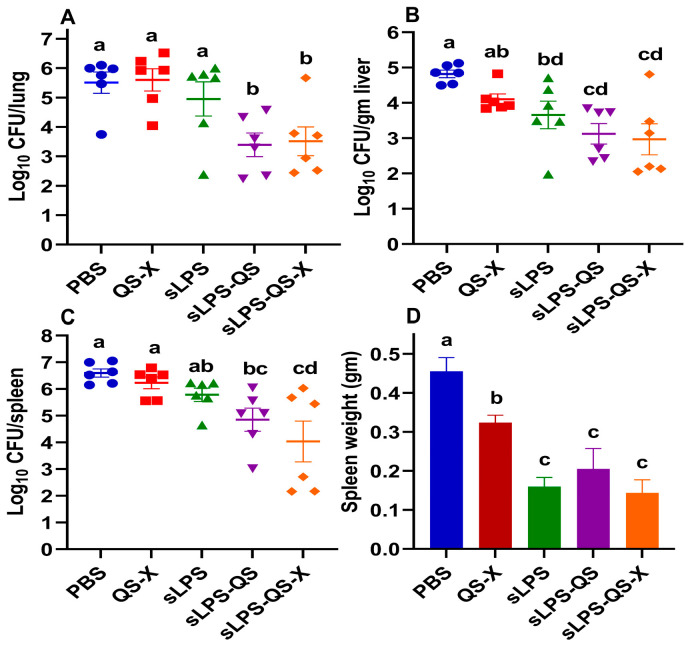
Impact of vaccine formulations on bioburdens of different organs and spleen weights one-month postchallenge. Data represent the calculated average Log ± SE, CFU of *B. abortus* S-19 recovered per lung (**A**), gram of liver (**B**), or spleen (**C**). (**D**) Spleen weights of mice. Animals challenged 4 weeks after the boost. *B. abortus* S19 was used at 1.92 × 10^7^ CFUs/mouse. Data were analyzed by ANOVA and Least Significant Difference (LSD) was used to determine differences between the groups. Different letters indicate that values are significantly different. A *p*-value of less than 0.05 was considered statistically significant.

The study analyzed the bacterial burden of *B. abortus* S19 in the lungs, livers, and spleens of mice one month after they were challenged with the bacteria. Comparison of *Brucella* burdens recovered from each experimental group revealed that the addition of QS or QS-X to sLPS vaccination significantly reduced bacterial burden in the lungs and spleens of the mice compared to sLPS vaccination alone (Figure 5A–C). Specifically, while sLPS alone did not significantly lower the lung bioburdens compared to PBS and Qs-X-vaccinated animals, a statistically significant reduction was found in the sLPS-QS and sLPS-QS-X groups relative to the unvaccinated group (PBS), which accounted for an approximate two log reduction each (Figure 5A). The addition of QS or QS-X to the sLPS vaccination resulted in a 36.24- or 27.56-fold reduction in lung bioloads compared to sLPS vaccination alone. We also observed a similar reduction in the liver bioburden for either groups that accounted for a 1.7- or 1.85-log reduction, respectively, relative to PBS (Figure 5B). LPS vaccination alone resulted in a significant bacterial reduction in the liver, however it appears that the addition of QS or QS-X to the sLPS vaccination resulted in a greater reduction in liver bioloads in mice than sLPS vaccination alone. Specifically, the use of QS or QS-X led to a 3.4- or 4.8-fold reduction in liver bioloads compared to sLPS vaccination alone (Figure 5B). In comparison to the PBS group, the sLPS-QS or sLPS-QS-X vaccination groups had a statistically significant reduction in bacteria in their spleens that accounted for 1.75- or 2.56-log reductions, respectively (Figure 5C). The study found that vaccination with sLPS alone did not cause a significant reduction in bacteria in the spleen compared to the PBS group, but the addition of QS or QS-X to the sLPS vaccination resulted in a significant reduction in bacteria in the spleen, with an 8.5- or 55.84-fold reduction, respectively, relative to the sLPS vaccination only (Figure 5C). Vaccination with sLPS also protected against splenomegaly as the spleens from mice who received the sLPS vaccination alone or in combination with QS or QS-X were statistically lower in weight than the spleens from mice in the PBS or QS-X groups (Figure 5D). Overall, the results suggest that the addition of QS or QS-X to sLPS vaccination may be an effective method for reducing bacterial burden in these organs and sLPS could prevent splenomegaly.

## 4. Discussion

*Brucella* LPS has been well characterized for decades and is known to have immunogenic properties [51,52]. It is considered non-pyrogenic and has a lower toxicity compared to other bacterial endotoxins [24]. These characteristics make it a promising candidate for use in vaccines as it is less likely to cause harmful side effects. It has been used in several preclinical studies to evaluate the efficacy of potential vaccine candidates against brucellosis [53,54,55,56]. However, as with any potential candidate, further research is needed to fully understand its utility and safety as a vaccine. 

In this research, we evaluated the efficacy of a safe subunit vaccine that combines *B. abortus* S19 LPS in tandem with saponin and/or xyloglucan in protecting against an intranasal *Brucella* challenge in BALB/C mice. The finding from this study showed that the tested formulations triggered robust local and systemic immune responses and enhanced protection in animals when they were challenged with an intranasal administration of S19. Specifically, we were able to recover both IgA and IgG1 but not IgG2a in the BALB/C mice bronchoalveolar lavages. While LPS alone was able to induce IgA production, the inclusion of saponin and/or xyloglucan along with the LPS was required to stimulate the production of a specific anti-*Brucella* IgG1 antibody in the respiratory mucus. sIgA is the key defense mechanism against foreign microorganisms at mucosal surfaces [57]; however, research has shown that IgG1 and IgG2a antibodies can also be secreted into the bronchoalveolar mucus of mice [58]. This suggests that these other antibodies may also contribute to the immune defense against foreign microorganisms in this specific location. In light of our finding that the sLPS-QS and sLPS-QS-X vaccines enhanced protection in the lungs of vaccinated animals, it is possible these antibodies may confer enhanced protection against *Brucella* challenge.

The tested vaccine was also able to induce a significant systemic antibody response in the combinations which included sLPS. Specifically, strong peripheral anti-LPS IgG1 and IgG2a responses were induced, representing Th1 and Th2 antibody isotypes, respectively, with a predominance of IgG1 over IgG2a in all the vaccinated groups. The sLPS-QS group produced detectable IgG2a levels after receiving a second dose of the vaccine with an enhanced IgG1 antibody response starting 14 days post-boosting that continued throughout the rest of the study, suggesting that the saponin may have played a role in directing the qualitative nature of the T helper cell response. 

Interestingly, we observed a biologically significant two-log *Brucella* reduction in the lung tissues of the LPS-QS- or LPS-QS-X-immunized mice relative to the unvaccinated group (PBS). Consistent with this, both groups had a significant decrease in their liver and splenic biological burdens. The addition of QS or QS-X to sLPS vaccination led to an enhanced reduction in bacterial burden in the lungs, livers, and spleens of the mice when compared to sLPS vaccination alone. That enhancement was likely due to saponin alone, as the addition of xyloglucan had a minimal effect on the bacterial burden in the three assayed tissues. Additionally, the inclusion of xyloglucan in the formulation led to a significant decrease in sIgA levels in the pulmonary mucus. A recent independent study showed that a nanovaccine containing Quillaja saponin and *Brucella abortus* S19 LPS elicited a mixed Th1/Th2 type of immunity in BALB/C mice when administered intranasally [27]. However, the study found a stronger IgG2a response than IgG1, which differed from the findings in the current study. The discrepancy could be due to differences in vaccine formulation and schedule, as well as the saponin dose used (100 *vs*. 15 µg/mouse). We speculate that the predominant isotype may be saponin-dose-dependent and the dose used in our study was not sufficient to polarize the immune response in BALB/C mice, which typically have a Th2-dominant response [59]. 

In the current study, we also observed a predominance of IgG1 over IgG2a in the LPS vaccination group only. Previous studies have found a mixed Th1/Th2 response with a predominant IgG1 when administering *B. melitensis* LPS-based vaccines intranasally [26] or trinitrophenyl-conjugated *B.abortus* LPS intraperitoneally to BALB/C mice [60]. This response was found to be directed specifically against the O-side chain [26]. These findings are consistent with the current study’s results.

Th1 response is crucial for effective defense against brucellosis, but an optimized Th2 response might also contribute to eradication [49]. The ability of B-cell epitope *Brucella* antigens to elicit effective humoral immunity has been demonstrated in the literature [61]. Our mucosal vaccine candidate was able to induce a persistent local and systemic immune response of Th1 and Th2 type. Perhaps, IgA and IgG1 in the sLPS-QS and sLPS-QS-X groups synergistically played a role against the extracellular *Brucellae* in the respiratory passage at the time of challenge that minimized the early dissemination of the invading pathogens. The same groups did exhibit a consistent reduction in bacterial titers in the lung and liver and spleen, indicating the indispensable role of adjuvants to achieve a generalized effect against the sLPS.

## 5. Conclusions 

The present study provides evidence that the proposed vaccine candidate can be safely administered, and evokes a strong specific local and systemic antibody response, as well as possibly a T cell response in the mouse model of brucellosis. The results show that while the vaccine does not provide sterilizing immunity, it leads to a partial protective immunity, including reduced bacterial tissue burdens, reduced inflammation as measured by reduced spleen size, and increased anti-LPS antibody titers. The study also suggests that inclusion of an adjuvant is necessary to achieve a generalized effect against the sLPS. However, further studies to optimize the vaccine formulation and dosage regimen are needed to further improve the efficacy of the vaccine. It is possible that the timing of the second dose was not optimal. The kinetics of the antibody response support this possibility as the titers of both IgG1 and especially IgG2a appear to still be in ascendancy at the time of the vaccine boost. A more thorough study on antibody kinetics and alternate, more optimized booster times may be required to resolve this issue. Additionally, because both LPS-dependent IgG1 and IgG2a isotype antibodies were induced differentially by saponin, an additional dose optimization of saponin may be required to maximize the antibody response.

## Data Availability

Data sharing not applicable.
